# Immunogenicity and Protective Efficacy of a Trivalent Whole-Virion Inactivated SARS-CoV-2 Vaccine Against Vaccine-Matched Ancestral, Delta, and Omicron BA.5 Variants in K18-hACE2 Mice

**DOI:** 10.3390/v18101092

**Published:** 2026-10-02

**Authors:** Hazel Yetiskin, Muhammet Ali Uygut, Shaikh Terkis Islam Pavel, Busra Kaplan, Aykut Ozdarendeli

**Affiliations:** 1 Vaccine Research and Development Institute, Erciyes University, 38280 Kayseri, Türkiye; hazelyetiskin@erciyes.edu.tr (H.Y.); mauygut@erciyes.edu.tr (M.A.U.); pavel@erciyes.edu.tr (S.T.I.P.); busrakaplan@erciyes.edu.tr (B.K.); 2 Vaccine Research, Development and Application Centre (ERAGEM), Erciyes University, 38280 Kayseri, Türkiye; 3 Department of Medical Microbiology, Faculty of Medicine, Erciyes University, 38280 Kayseri, Türkiye

**Keywords:** SARS-CoV-2, trivalent vaccine, inactivated vaccine, Omicron BA.5, Delta, neutralizing antibodies, pandemic preparedness

## Abstract

Background: Continuous antigenic evolution of SARS-CoV-2 has reduced the effectiveness of vaccines based on a single ancestral strain, highlighting the need for multivalent vaccine strategies. We developed a trivalent whole-virion inactivated vaccine containing ancestral (ERAGEM), Delta, and Omicron BA.5 antigens and evaluated its immunogenicity and protective efficacy in K18-hACE2 transgenic mice. Methods: β-propiolactone-inactivated ERAGEM, Delta, and Omicron BA.5 antigens were combined at 1 µg each (3 µg total) with aluminium hydroxide and administered intraperitoneally on days 0 and 21. Variant-specific binding and neutralizing antibody responses were assessed by enzyme-linked immunosorbent assay (ELISA) and microneutralization assays. On day 42, mice were challenged with ERAGEM, Delta, or Omicron BA.5. Survival, body weight, body temperature, infectious viral titers, and viral RNA loads were evaluated. Results: The trivalent vaccine induced binding and neutralizing antibodies against all three vaccine components, and titers rose after the booster dose. Neutralizing responses were highest against the BA.5 component. Vaccinated mice showed 100% survival following challenge with each vaccine-matched variant and maintained stable body weight and temperature throughout the observation period. Unvaccinated controls challenged with Omicron BA.5 showed no mortality, so protection against this component could be assessed only virologically. No infectious virus was detected in the lungs of vaccinated animals, while viral titers and RNA loads in the nasal turbinates were significantly reduced compared with unvaccinated controls. Conclusions: A trivalent whole-virion inactivated SARS-CoV-2 vaccine containing ancestral, Delta, and Omicron BA.5 antigens elicited variant-specific humoral immunity and protected mice against challenge with each vaccine-matched component: against lethal challenge for the ancestral and Delta components, and against viral burden for Omicron BA.5, which was not lethal in unvaccinated controls. These outcomes were obtained at a lower per-antigen dose than in our previously reported monovalent and bivalent formulations; because those were independent studies rather than contemporaneous comparators, this suggests a dose-sparing potential that warrants formal evaluation rather than demonstrating one. Cross-protection against variants not included in the formulation was not assessed.

## 1. Introduction

Since its emergence in late 2019, SARS-CoV-2 has caused unprecedented global morbidity and mortality and continues to circulate as an evolving pathogen. The vaccines that received emergency authorization in 2020 were designed against the spike protein of the ancestral Wuhan strain and were highly effective at preventing severe disease in the early phase of the pandemic [1]. Their performance, however, has been progressively undermined by the appearance of antigenically distinct variants. Successive waves driven by the Delta (B.1.617.2) and Omicron lineages demonstrated that mutations concentrated in the spike receptor-binding domain and N-terminal domain can substantially reduce the neutralizing activity of antibodies elicited by prior infection or ancestral-strain vaccination [2,3]. The Omicron sublineage BA.5, in particular, combined enhanced transmissibility with pronounced immune escape and drove one of the largest global infection waves [3]. This pattern of antigenic drift has made clear that vaccine antigens matched to a single, often outdated, strain offer incomplete and short-lived population protection.

One strategy to counter this moving target is multivalent vaccination, in which antigens from two or more variants are combined within a single formulation in order to cover several antigenically distinct strains at once. It should be noted that such an approach is inherently reactive rather than anticipatory: it can only cover variants that are already known and have been incorporated as components, and it does not constitute a universal or pan-variant vaccine. The evidence that multivalency broadens protection is also mixed, since immune imprinting toward historical strains may blunt responses to newer components [4]. The bivalent mRNA boosters encoding the ancestral spike together with an Omicron spike (BA.1 or BA.4/5) provided the first large-scale demonstration of this principle in humans [5]. Since then, trivalent and multivalent SARS-CoV-2 designs have been pursued across essentially every vaccine platform, including mRNA [6], recombinant spike or receptor-binding-domain (RBD) subunit antigens [7], virus-like particles [8], and viral-vectored constructs [9], with some efforts extending the concept toward “universal” sarbecovirus or pan-betacoronavirus coverage [10,11]. Across platforms, these studies consistently report that combining antigens broadens cross-neutralization (and, where tested, cross-protection) without abrogating responses to the individual components, reinforcing multivalency as a rational route to variant-resilient immunity.

Among the available platforms, whole-virion inactivated vaccines occupy a distinct and practically valuable niche. Because the antigen is the intact, inactivated virion, the immune system is presented with the full complement of structural proteins (spike, nucleocapsid, membrane, and envelope) rather than spike alone, which may broaden the targeted epitope repertoire [12]. Inactivated vaccines are produced through well-characterized, scalable processes, have a long-established safety record, and are stable under standard cold-chain conditions, making them particularly suited to rapid deployment and to settings with limited infrastructure [13]. Our group previously developed the ancestral-strain inactivated vaccine ERUCoV-VAC, which protected mice and ferrets and was subsequently licensed and deployed as TURKOVAC [12,13,14]. Building on this platform, we recently reported bivalent inactivated formulations pairing the ancestral strain (ERAGEM) with either the Delta or the Omicron (BA.5) variant, both of which elicited broadened neutralizing responses and protection against challenge [15]. The feasibility of trivalent inactivated coronavirus vaccines more broadly is illustrated in veterinary medicine, where a trivalent inactivated formulation against the porcine enteric coronaviruses PEDV, TGEV, and PDCoV recently conferred protection against each component virus in piglets [16]. For SARS-CoV-2, however, multivalent inactivated formulations remain comparatively rare. An inactivated trivalent vaccine combining an ancestral strain with the Delta and an earlier Omicron (BA.1) variant has been reported to broaden neutralizing-antibody responses in mice [17], yet inactivated designs incorporating the later, markedly immune-evasive Omicron BA.5 sublineage, and in particular, their protective efficacy against challenge with each component variant, have not, to our knowledge, been characterized.

An important concern in multivalent vaccine design is whether combining multiple antigens compromises responses to individual components, and whether coverage of more variants can be achieved without escalating the total antigen content. Here, we developed a trivalent whole-virion inactivated vaccine co-formulating locally isolated ERAGEM, Delta, and Omicron (BA.5) antigens and evaluated its immunogenicity and protective efficacy in K18-hACE2 transgenic mice. Each antigen was included at 1 µg per dose, below the per-antigen amounts used for our monovalent (3–6 µg) and bivalent (1.5 µg) formulations, allowing us to test whether comprehensive, variant-specific protection can be maintained under a dose-sparing strategy. Beyond characterizing variant-specific binding and neutralizing antibody responses, we determined survival, clinical parameters, and respiratory-tract viral burden following homologous challenge with each of the three component variants, including lethal challenge with ERAGEM and Delta, thereby providing protective-efficacy data for an inactivated multivalent formulation that includes the BA.5 sublineage.

## 2. Materials and Methods

### 2.1. Cells and Viruses

Vero E6 cells (African green monkey kidney; ATCC CRL-1586) were maintained in Dulbecco’s modified Eagle’s medium (DMEM) supplemented with 10% heat-inactivated fetal bovine serum (FBS) and L-glutamine (Sigma-Aldrich, Darmstadt, Germany) at 37 °C under 5% CO_2_. The three SARS-CoV-2 isolates used in this study, namely the ancestral strain (designated ERAGEM), the Delta (B.1.617.2) variant, and the Omicron BA.5 sublineage, were recovered from nasopharyngeal specimens of COVID-19 patients admitted to Kayseri City Training and Research Hospital, following the procedures established for our inactivated vaccine platform [12,15]. Specimens collected in viral transport medium were inoculated onto Vero E6 monolayers, adsorbed for 1 h at 37 °C, and then overlaid with DMEM (Sigma-Aldrich, St. Louis, MO, USA) containing 2% FBS (Gibco, Thermo Fisher Scientific, Grand Island, NY, USA) and observed for cytopathic effect (CPE). All propagation and titration of infectious virus was performed under enhanced biosafety level 3 (BSL-3) containment.

### 2.2. Virus Titration

Infectious titers were determined by a 50% tissue culture infectious dose (TCID50) assay. Vero E6 cells were seeded in 96-well plates (Greiner Bio-One GmbH, Frickenhausen, Germany) at 1.5 × 10^4^ cells per well and incubated for 24 h. Ten-fold serial dilutions of each viral stock were added in triplicate (50 µL per well); after a 1 h adsorption period the inoculum was replaced with DMEM (Sigma-Aldrich, St. Louis, MO, USA) containing 2% FBS (Gibco, Thermo Fisher Scientific, Grand Island, NY, USA), and the plates were incubated for five days together with uninfected control wells. Wells in which more than 25% of the monolayer exhibited CPE were scored as positive, and TCID50/mL values were calculated using the Spearman–Kärber method.

### 2.3. Preparation of Inactivated Vaccine Antigens

Whole-virion antigens for each variant were produced and inactivated as previously established for our inactivated SARS-CoV-2 vaccine platform [12,15]. In brief, Vero E6 cells grown in a cell-factory system were infected with the respective variant at a low multiplicity of infection. Culture supernatants were harvested, clarified by centrifugation, and inactivated with β-propiolactone (Sigma-Aldrich, St. Louis, MO, USA); complete inactivation was verified by the absence of CPE upon serial passage in Vero E6 cells. The inactivated virus was concentrated with polyethylene glycol (Promega Corporation, Madison, WI, USA) and further purified through a sucrose (Sigma-Aldrich, St. Louis, MO, USA) density gradient, after which the total antigen content was quantified by the Lowry method. The purified, inactivated whole-virion antigens were then adsorbed to aluminium hydroxide (InvivoGen, San Diego, CA, USA) (250 µg per dose) for formulation.

### 2.4. Vaccine Formulation, Immunization, and Challenge

**Figure 1:**
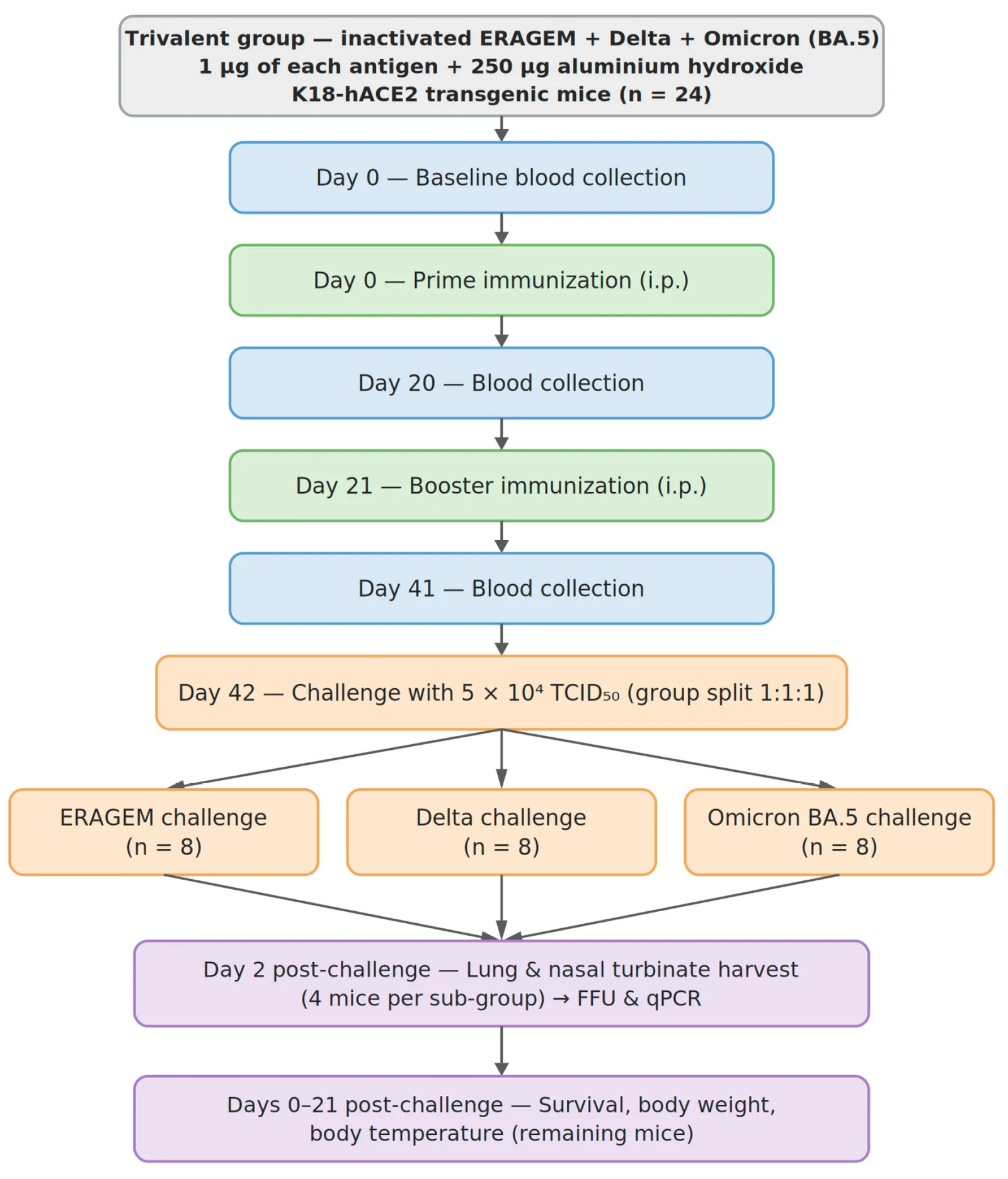
Experimental design for the trivalent vaccine study. K18-hACE2 transgenic mice (*n* = 24) received a trivalent inactivated vaccine (1 µg each of ERAGEM, Delta, and Omicron BA.5 antigens with aluminium hydroxide) as a prime (day 0) and a booster (day 21) by the intraperitoneal route, with blood collected on days 0, 20, and 41. On day 42, the cohort was split 1:1:1 and challenged with ERAGEM, Delta, or Omicron (BA.5) at 5 × 10^4^ TCID50 (*n* = 8 per sub-group). Lungs and nasal turbinates were harvested from four mice per sub-group on day 2 post-challenge for fluorescent focus assay and quantitative RT-PCR (qPCR); the remaining mice were monitored for survival, body weight, and body temperature for 21 days. Unvaccinated, PBS-treated mice were challenged in parallel as negative controls, with three control mice per variant euthanized on day 2 for FFU and qPCR.

The trivalent vaccine was prepared by combining the inactivated ERAGEM, Delta, and Omicron (BA.5) antigens at 1 µg of each antigen per dose (3 µg total antigen), adsorbed to 250 µg aluminium hydroxide (InvivoGen, San Diego, CA, USA). K18-hACE2 (human angiotensin-converting enzyme 2) transgenic mice (B6.Cg-Tg(K18-ACE2)2Prlmn/J; The Jackson Laboratory, Bar Harbor, ME, USA) were immunized intraperitoneally with a prime dose on day 0 and a booster dose on day 21. Blood was collected before immunization (day 0) to establish baseline titers, on day 20 to assess the post-prime response, and on day 41 to assess the post-boost response. On day 42, the immunized cohort (*n* = 24) was divided into three challenge sub-groups of 8 mice each and inoculated with the ERAGEM, Delta, or Omicron (BA.5) variant at 5 × 10^4^ TCID50; unimmunized, PBS-treated mice challenged in parallel served as negative controls. Two days after challenge, 4 mice per vaccinated sub-group and 3 mice per unimmunized control group were euthanized for quantification of infectious viral titer and viral RNA load in lung and nasal turbinate tissue, and the remaining 4 mice per vaccinated sub-group were monitored daily for 21 days for survival, body weight, and body temperature. The experimental timeline is summarized in Figure 1.

### 2.5. Endpoint ELISA

Variant-specific binding antibody titers were measured by endpoint ELISA. MaxiSorp plates (Thermo Fisher Scientific, Roskilde, Denmark) were coated overnight at 4 °C with the corresponding inactivated variant antigen (1 µg per well) in carbonate buffer (pH 9.4), so that ERAGEM, Delta, and Omicron (BA.5) antigens were used to detect the response to each respective component. Serum samples were diluted two-fold starting at 1:100, applied to the plates, and incubated at 37 °C for 1 h. After washing, the bound antibody was detected using horseradish-peroxidase-conjugated goat anti-mouse IgG (Southern Biotech, Birmingham, AL, USA; 1:2000), developed with TMB substrate (Thermo Scientific, Rockford, IL, USA), and stopped with 2 M sulfuric acid (Sigma-Aldrich, St. Louis, MO, USA) before reading the optical density at 450 nm. The endpoint titer was defined as the highest serum dilution yielding a signal above a cut-off set at three times the background, and results were expressed as geometric mean titers (GMTs) ± standard error. 

### 2.6. Microneutralization Test

Neutralizing antibody titers were determined by microneutralization assay. Heat-inactivated sera (56 °C, 1 h) were serially diluted two-fold and incubated with 100 TCID50 of the corresponding SARS-CoV-2 variant for 1 h at 37 °C. The serum–virus mixtures were transferred onto confluent Vero E6 monolayers in 96-well plates (Greiner Bio-One GmbH, Frickenhausen, Germany), with virus-only and cell-only wells included as controls, and CPE was scored 3–4 days post-infection. The 50% neutralization titer (MN50/NT50) was taken as the highest serum dilution that prevented CPE in half of the replicate wells, and a titer of ≥1:8 was regarded as seropositive.

### 2.7. Fluorescent Focus Assay

Infectious virus in tissues was quantified by fluorescent focus assay [18]. Lung and nasal turbinate samples were homogenized in DMEM (Sigma-Aldrich, St. Louis, MO, USA) containing penicillin and streptomycin (Sigma-Aldrich, St. Louis, MO, USA) and clarified by centrifugation (18,000× *g*, 10 min). Clarified supernatants were inoculated in triplicate onto Vero E6 monolayers; after 1 h of adsorption, the cells were washed and overlaid with medium containing 1% carboxymethyl cellulose (Sigma-Aldrich, St. Louis, MO, USA), then incubated for 72 h. Monolayers were fixed, permeabilized, blocked, and stained with a human anti-SARS-CoV-2 nucleocapsid antibody (GenScript, Piscataway, NJ, USA; 1:2500) followed by an FITC-conjugated goat anti-human IgG (Southern Biotech, Birmingham, AL, USA; 1:1000). Fluorescent foci were enumerated by immunofluorescence microscopy and expressed as fluorescent focus units per millilitre (FFU/mL).

### 2.8. Quantitative RT-PCR

Viral RNA loads were measured from the same clarified tissue homogenates. RNA was extracted using the QIAamp Viral RNA Mini Kit (QIAGEN, Hilden, Germany) and SARS-CoV-2 RNA was quantified with the Diagnovital SARS-CoV-2 Real-Time PCR Kit v2.0 (RTA Laboratories, Istanbul, Türkiye) on a Rotor-Gene Q instrument (QIAGEN, Hilden, Germany). Absolute copy numbers were derived from a standard curve generated with ten-fold dilutions of SARS-CoV-2 RNA standards of known concentration, and results were expressed as RNA copies per millilitre (copies/mL).

### 2.9. Statistical Analysis

Analyses were performed in GraphPad Prism v10 (GraphPad Software, San Diego, CA, USA). Survival was depicted with Kaplan–Meier curves and compared by the log-rank test. Prime-versus-boost comparisons of endpoint ELISA and microneutralization (MN50) titers were made with the paired Student’s *t*-test, as both measurements came from the same animals; comparisons of FFU and RNA copy numbers between vaccinated and control groups used the unpaired Student’s *t*-test. Statistical significance was defined as * *p* < 0.05, ** *p* < 0.01, *** *p* < 0.001, and **** *p* < 0.0001, as indicated in the figures. A formal test of normality (for example, the Shapiro–Wilk test) was not performed; with three to four animals per group, such tests have very low power, and we did not consider them informative. Comparisons were made with Student's *t*-test, as in our previous challenge studies on the same platform, and no correction for multiple comparisons was applied, the analyses being confined to a small number of pre-specified prime-versus-boost and vaccinated-versus-control comparisons. We acknowledge that the distributional assumptions of the parametric tests could not be verified at these group sizes and that no adjustment was made for multiplicity. Individual animal values are therefore shown for every group, and the statistical comparisons should be read as supportive of the differences described rather than as definitive. Given the limited sample sizes imposed by the BSL-3 challenge model, individual data points are shown for all groups, and the statistical comparisons should be read as supportive rather than definitive.

### 2.10. Ethics Statement

All animal procedures were reviewed and approved by the Erciyes University Animal Experiments Local Ethics Committee (EUHADYEK; approval no. 22/144, approved 17 May 2022) and by the Committee for Animal Biosafety Level 3 Research (ERAGEM; protocol no. IP-3-14, approved 26 June 2022), and were performed under enhanced biosafety level 3 containment in accordance with the relevant institutional and national guidelines for the care and use of laboratory animals. No a priori power calculation was performed. Group sizes were set to match those used in our previous monovalent and bivalent challenge studies in the same model, in which the effect sizes for survival and for reduction in viral burden were large, and were constrained by the requirements of high-containment BSL-3 work and by the ethical principle of reduction. The study is therefore powered to detect large effects only; this limitation is acknowledged in Section 4.

## 3. Results

### 3.1. Survival Following Challenge with Vaccine-Matched Variants

**Figure 2:**
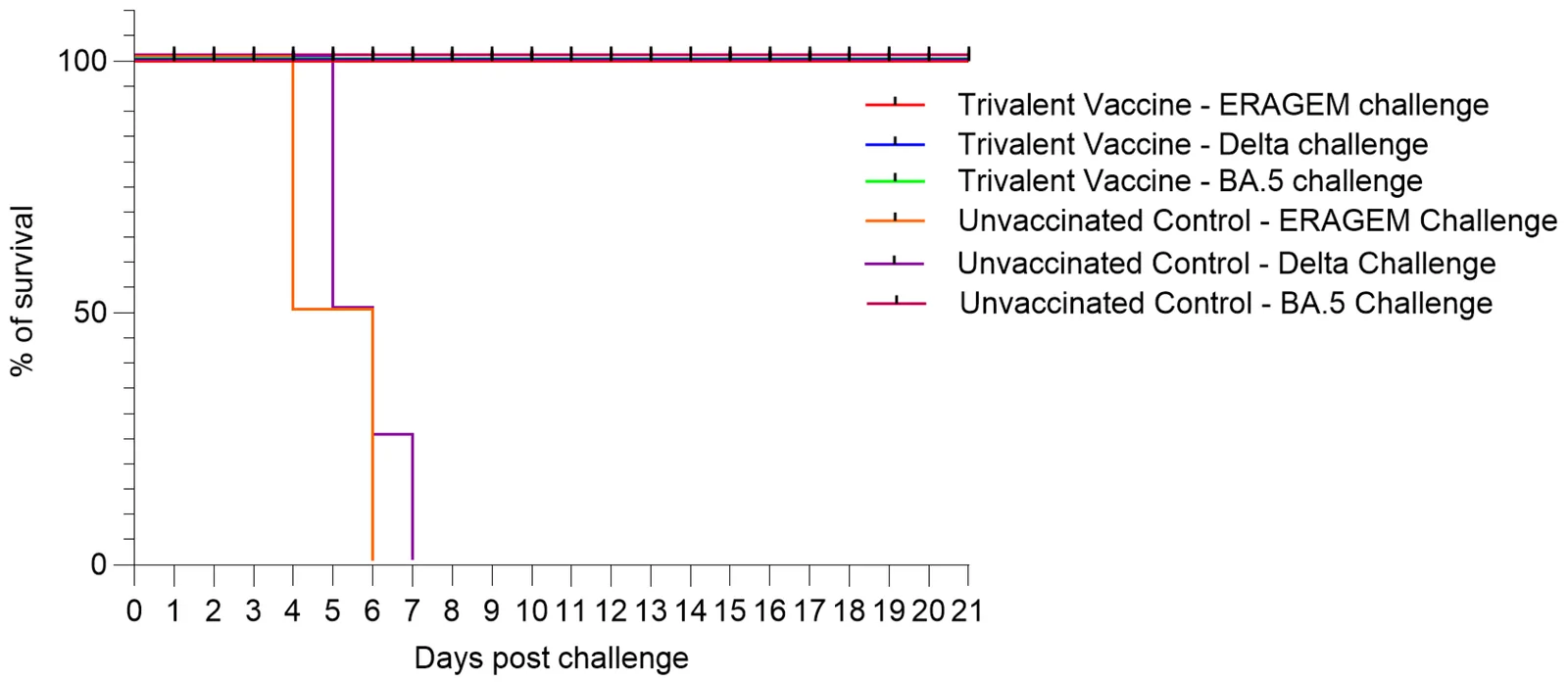
Survival following challenge with vaccine-matched variants. Kaplan–Meier curves show the percentage of surviving mice over 21 days post-challenge. “Trivalent vaccine” denotes the inactivated ERAGEM + Delta + Omicron BA.5 formulation. Trivalent-vaccinated groups challenged with ERAGEM (red), Delta (blue), or Omicron BA.5 (green) maintained 100% survival, whereas unvaccinated controls challenged with ERAGEM (orange) or Delta (purple) reached complete mortality by days 6 and 7, respectively; the unvaccinated Omicron BA.5 control (magenta) showed no mortality. Survival was monitored in the mice remaining after the day-2 tissue harvest (4 vaccinated mice per sub-group).

Following challenge on day 42, every trivalent-vaccinated mouse survived the entire 21-day observation period, irrespective of the challenge variant, corresponding to 100% survival in each of the ERAGEM, Delta, and Omicron (BA.5) sub-groups (Figure 2). The unvaccinated control groups, by contrast, succumbed to the more virulent variants: animals challenged with ERAGEM or Delta reached complete mortality by days 6 and 7, respectively. Controls challenged with Omicron (BA.5) showed no mortality, consistent with the attenuated virulence of this sublineage in the K18-hACE2 model in the absence of vaccination.

### 3.2. Vaccinated Mice Maintained Stable Body Weight and Temperature

**Figure 3:**
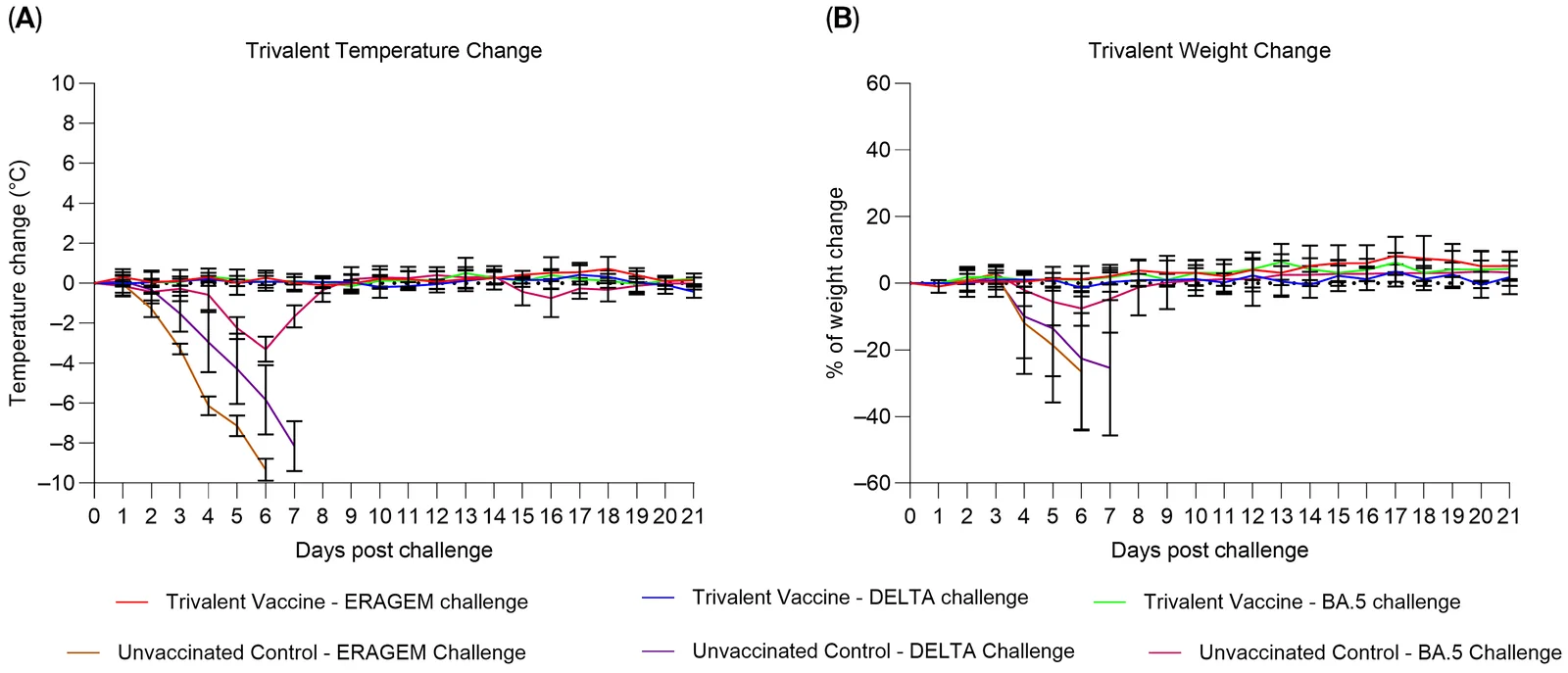
Body temperature and weight changes after challenge. Mean change (±SEM) in (**A**) body temperature (°C) and (**B**) body weight (%) over 21 days post-challenge. “Trivalent vaccine” denotes the inactivated ERAGEM + Delta + Omicron BA.5 formulation. Trivalent-vaccinated mice challenged with ERAGEM (red), Delta (blue), or Omicron BA.5 (green) remained stable, whereas unvaccinated ERAGEM (orange) and Delta (purple) controls developed pronounced hypothermia and weight loss peaking on days 5–7; the unvaccinated Omicron BA.5 control (magenta) remained comparatively stable.

Across all three challenges, vaccinated mice maintained stable body temperature and body weight throughout the observation period, with only minor day-to-day fluctuations (Figure 3A,B). The unvaccinated ERAGEM and Delta control groups, in contrast, developed pronounced hypothermia and progressive weight loss from approximately day 2, reaching their nadir between days 5 and 7 in parallel with the onset of mortality. The Omicron (BA.5) control group remained comparatively stable, mirroring its survival outcome and reinforcing the observation of the lower virulence of this sublineage in this model.

### 3.3. Booster Immunization Elicited Strong Variant-Specific Binding Antibodies

**Figure 4:**
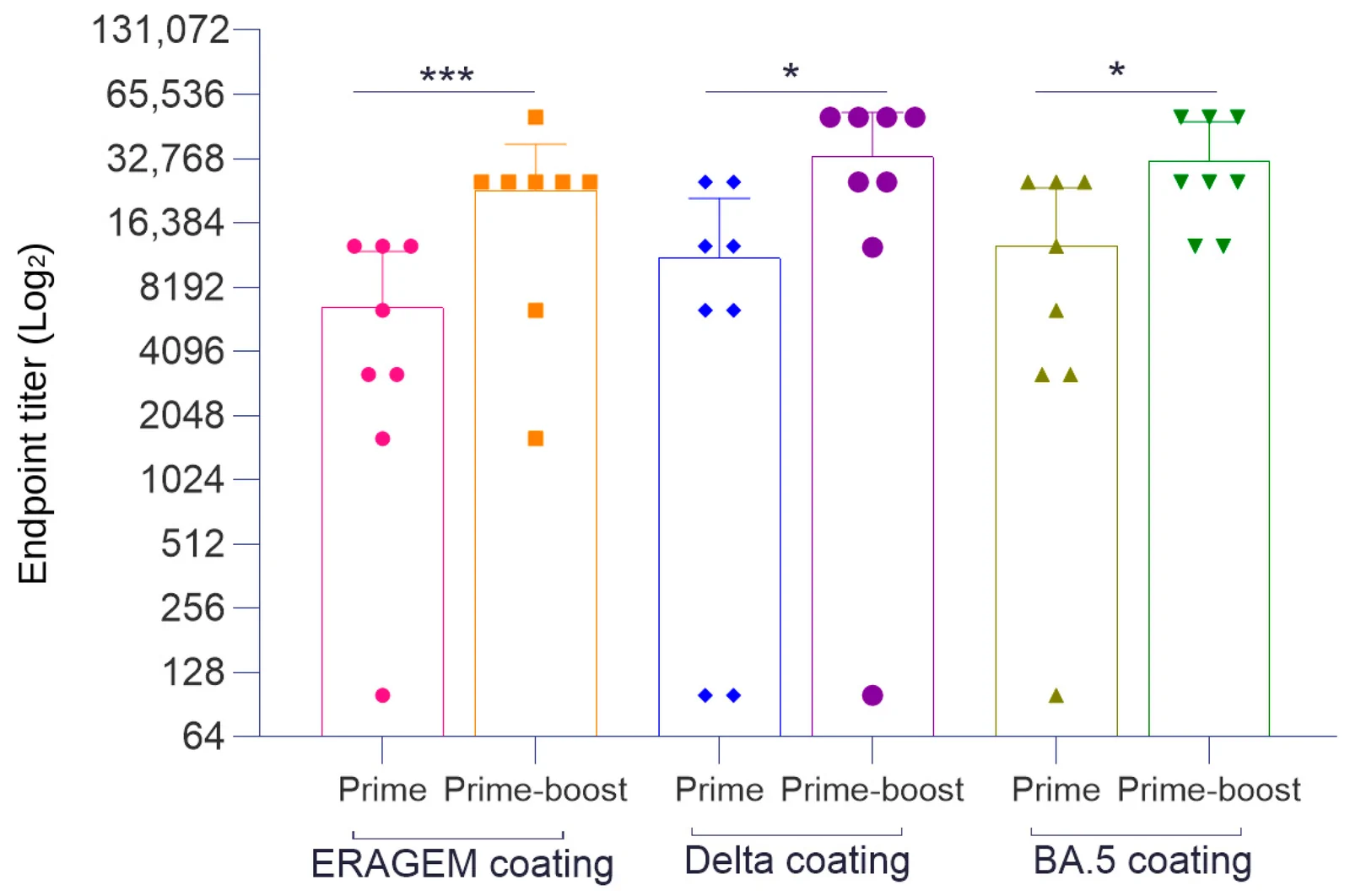
Variant-specific endpoint ELISA titers after prime and booster immunization. Endpoint binding-antibody titers (log_2_) in trivalent-vaccinated mice, measured on plates coated with the homologous antigen (ERAGEM, Delta, or Omicron BA.5), after the prime and the booster dose. Bars show geometric mean titers and each symbol represents one mouse. * *p* < 0.05, *** *p* < 0.001 (paired *t*-test); analyses in GraphPad Prism v10. Symbols denote individual mice: ERAGEM, pink circles (prime) and orange squares (prime–boost); Delta, blue diamonds (prime) and purple circles (prime–boost); BA.5, olive upward triangles (prime) and green downward triangles (prime–boost).

Endpoint ELISA titers against each component antigen rose from the prime to the post-boost time point (Figure 4). The GMTs rose from 3489.6 to 16,599.5 for ERAGEM, from 5868.8 to 27,916.9 for Delta, and from 3805.4 to 16,566.9 for Omicron (BA.5); the increase was most significant for the ERAGEM component (*p* < 0.001), with significant rises also observed against Delta and Omicron BA.5 (*p* < 0.05). The booster therefore enhanced binding antibodies against all three variants at once, with no component left without a measurable boost.

### 3.4. The Trivalent Vaccine Induced Neutralizing Antibodies Against All Three Variants

**Figure 5:**
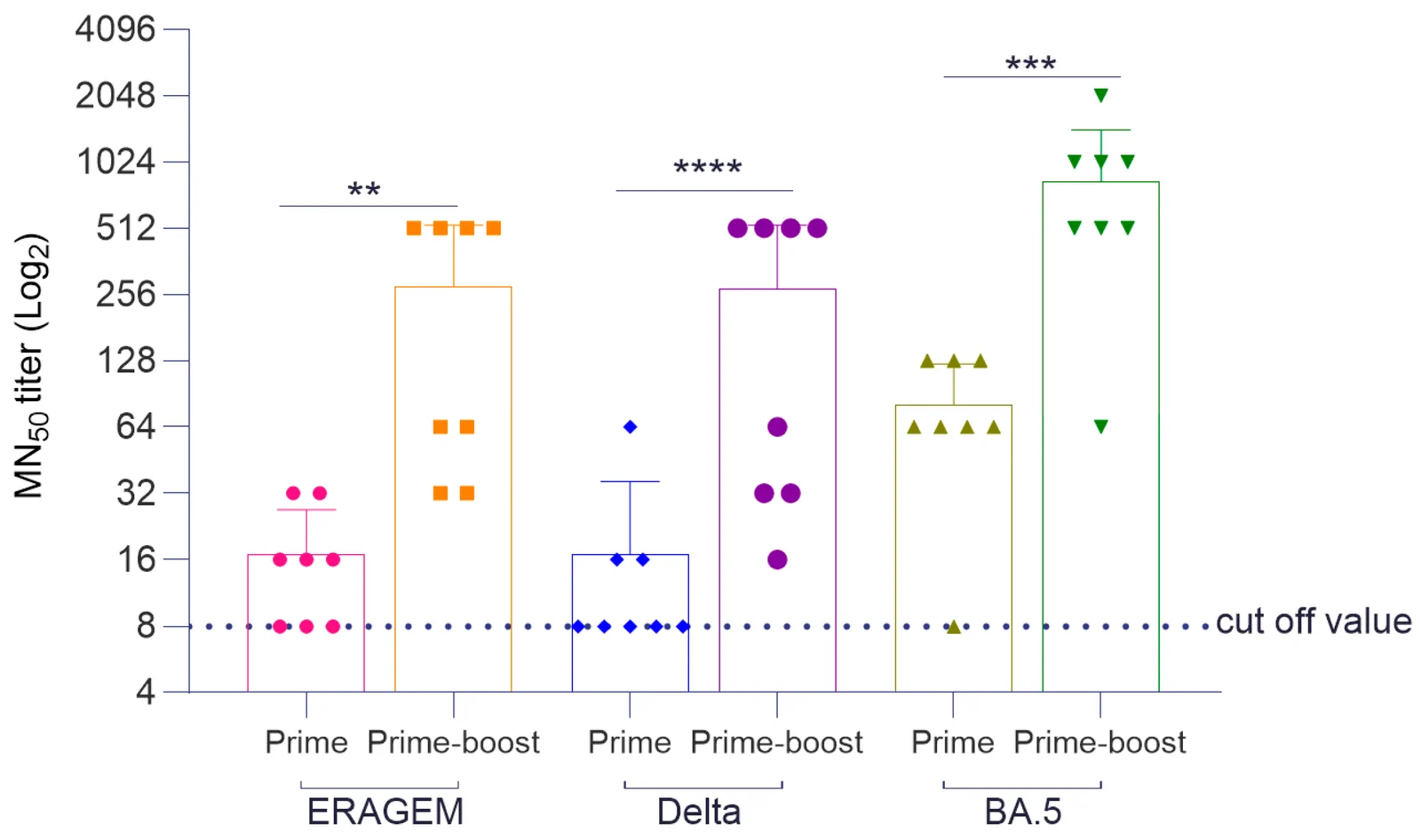
Variant-specific neutralizing antibody titers (MN50). MN50 titers (log_2_) against ERAGEM, Delta, and Omicron BA.5 after the prime and the booster dose. Bars show geometric means, each symbol represents one mouse, and the dotted line marks the assay cut-off (1:8). ** *p* < 0.01, *** *p* < 0.001, **** *p* < 0.0001 (paired *t*-test). Symbols denote individual mice: ERAGEM, pink circles (prime) and orange squares (prime–boost); Delta, blue diamonds (prime) and purple circles (prime–boost); BA.5, olive upward triangles (prime) and green downward triangles (prime–boost).

Neutralizing antibody titers, measured by microneutralization assay, likewise increased from prime to boost against each variant (Figure 5). The MN50 GMTs rose from 14.6 to 152.2 for ERAGEM (*p* < 0.01), from 12.3 to 128.0 for Delta (*p* < 0.0001), and from 64.0 to 609.8 for Omicron (BA.5) (*p* < 0.001). The most pronounced neutralizing response was directed against the BA.5 component, indicating that inclusion of the BA.5 antigen elicited robust sublineage-specific neutralization rather than being outcompeted by the ancestral and Delta antigens.

### 3.5. Vaccination Rendered Pulmonary Infectious Virus Undetectable and Reduced Nasal Turbinate Titers

**Figure 6:**
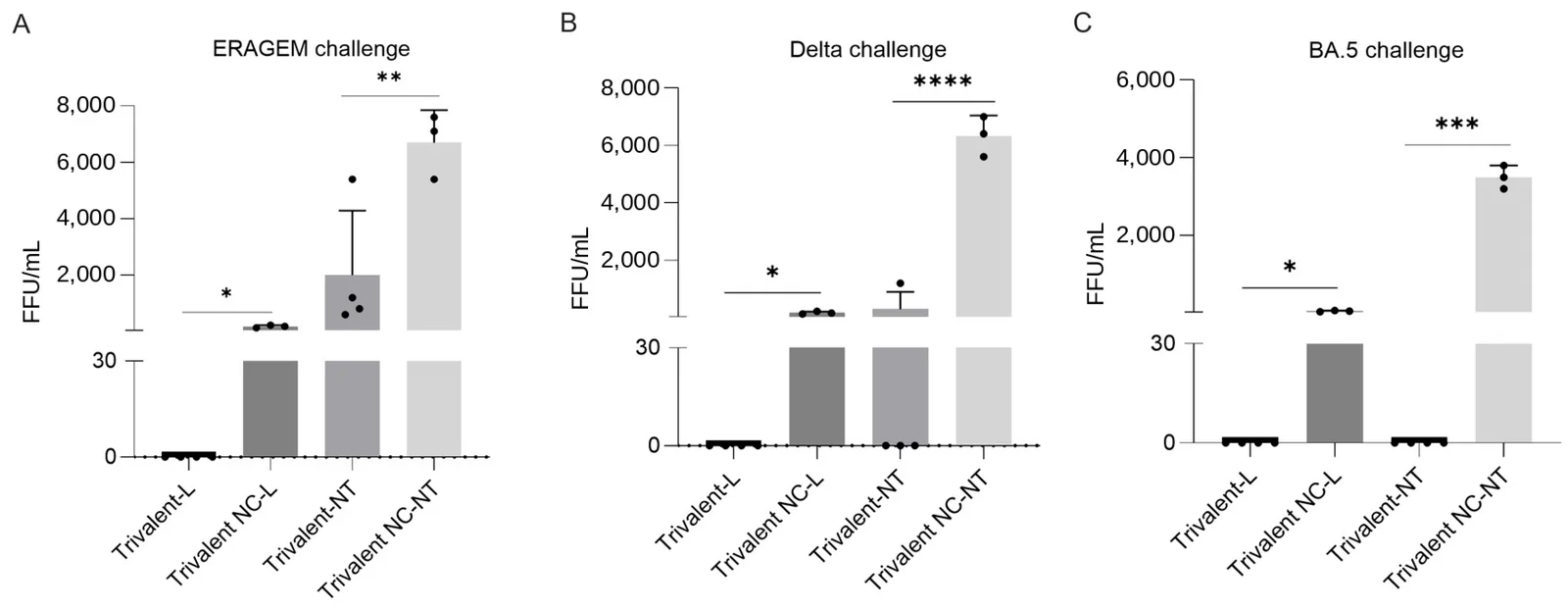
Infectious viral titers in respiratory tissues two days post-challenge (FFU). Fluorescent focus units per millilitre (FFU/mL) in lung (L) and nasal turbinate (NT) of trivalent-vaccinated mice and unimmunized negative controls (NCs) after (**A**) ERAGEM, (**B**) Delta, and (**C**) Omicron BA.5 challenge. Trivalent-L and Trivalent-NT, vaccinated lung and nasal turbinate; Trivalent NC-L and Trivalent NC-NT, unvaccinated control lung and nasal turbinate. Bars show geometric means and each symbol represents one mouse (*n* = 4 vaccinated and *n* = 3 control mice per challenge). * *p* < 0.05, ** *p* < 0.01, *** *p* < 0.001, **** *p* < 0.0001 (unpaired *t*-test).

Infectious viral titers in respiratory tissues were quantified by fluorescent focus assay two days post-challenge (Figure 6A–C). In the lungs of vaccinated mice, infectious virus was below the limit of detection in all three challenge sub-groups, whereas the corresponding unvaccinated controls had measurable pulmonary titers of 164.5, 129.7, and 38.2 FFU/mL for the ERAGEM, Delta, and Omicron (BA.5) challenges, respectively. In the nasal turbinates, vaccinated mice showed markedly lower titers than controls: 725.2 versus 6928.2 FFU/mL (ERAGEM), 385.2 versus 6060.2 FFU/mL (Delta), and below the limit of detection versus 3074.2 FFU/mL (Omicron BA.5) (*p* < 0.05 to *p* < 0.0001). Vaccination thus rendered infectious virus undetectable in the lungs and substantially reduced infectious viral titers in the nasal turbinates for every component variant.

### 3.6. Viral RNA Loads Were Markedly Reduced in Vaccinated Mice

**Figure 7:**
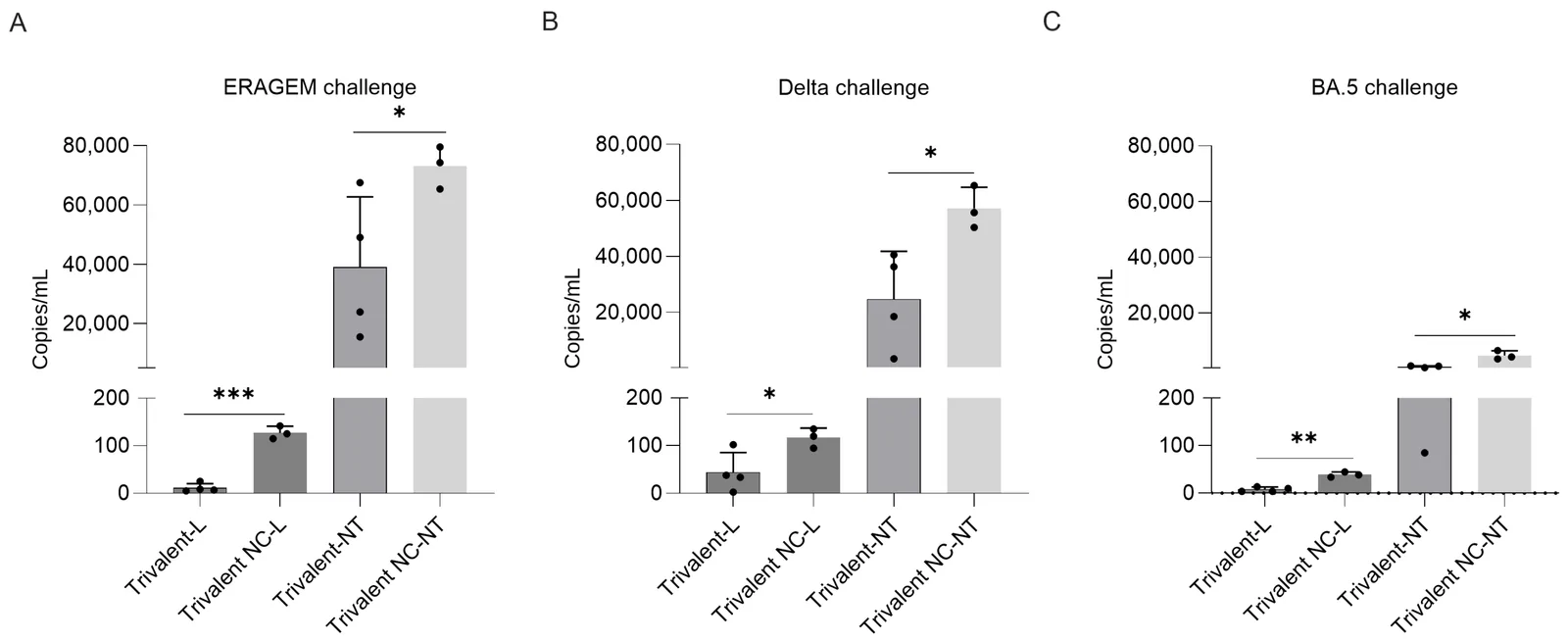
Viral RNA copy numbers in respiratory tissues two days post-challenge (qPCR). Viral RNA copies per millilitre (copies/mL) in lung (L) and nasal turbinate (NT) of trivalent-vaccinated mice and unimmunized negative controls (NCs) after (**A**) ERAGEM, (**B**) Delta, and (**C**) Omicron BA.5 challenge; labels as in Figure 6. Bars show geometric means and each symbol represents one mouse (*n* = 4 vaccinated and *n* = 3 control mice per challenge). * *p* < 0.05, ** *p* < 0.01, *** *p* < 0.001 (unpaired *t*-test).

Viral RNA copy numbers, determined by quantitative RT-PCR, were consistent with the infectious-titer data (Figure 7A–C). In the lungs, vaccinated mice harboured 13.6, 12.5, and 12.2 copies/mL after ERAGEM, Delta, and Omicron (BA.5) challenge, compared with 122.4, 111.4, and 40.1 copies/mL in the respective controls. In the nasal turbinates, vaccinated mice carried 8924.1, 17,007.1, and 158.2 copies/mL versus 70,254.2, 58,214.1, and 4420.1 copies/mL in controls (*p* < 0.05 to *p* < 0.001). The lowest absolute RNA loads were again observed in the Omicron (BA.5) challenge, in keeping with the reduced virulence of this sublineage. Across all three variants, the vaccine sharply reduced viral burden in both the lower and upper airway.

### 3.7. Comparable, Variant-Specific Responses to All Three Antigens

Because antigenic competition can blunt responses to individual components of a multivalent vaccine, we examined whether any of the three antigens was disadvantaged at the reduced per-antigen dose of 1 µg. Each component elicited a significant booster response in both binding and neutralizing antibodies, and all three challenge sub-groups showed 100% survival, with infectious virus undetectable in the lungs (Table 1); for the BA.5 sub-group, where the challenge was not lethal in unvaccinated controls, this survival cannot be read as protection against lethal disease. The neutralizing response to the Omicron (BA.5) component was in fact the strongest of the three, and no component was left without a measurable boost, with no apparent loss of component-specific responses under the conditions tested. Each of these responses, and the protection that accompanied them, was elicited with only 1 µg of each antigen per dose. The monovalent and bivalent formulations referred to for comparison were evaluated in separate, independent studies rather than as contemporaneous comparator groups, so any dose-related inference drawn from this comparison is exploratory.

## 4. Discussion

We first set out what the present study does and does not establish. This study provides the following new evidence: (i) a whole-virion inactivated formulation containing three antigenically distinct SARS-CoV-2 antigens, including the immune-evasive Omicron BA.5 sublineage, elicits variant-specific binding and neutralizing antibodies against each of its components; (ii) it protects K18-hACE2 mice against lethal challenge with the ancestral and Delta components and reduces viral burden in the lower and upper respiratory tract after challenge with each vaccine-matched component, including BA.5; and (iii) these outcomes were obtained with 1 µg of each antigen per dose, below the per-antigen amounts used in our previous monovalent and bivalent formulations, which suggests a dose-sparing potential.

The following are not addressed by this study, and no claim is made regarding them: (i) cross-protection or cross-neutralization against variants not included in the formulation, including contemporary sublineages such as XBB.1.5 and JN.1, which were not tested; (ii) any incremental benefit of the trivalent formulation relative to monovalent or bivalent formulations, since contemporaneous lower-valency control groups were not included; (iii) formal dose-titration, which was not performed, so the dose-sparing observation is exploratory; (iv) protection against lethal challenge with Omicron BA.5, which could not be assessed because this sublineage caused no mortality in unvaccinated K18-hACE2 controls; and (v) the durability of the responses, since long-term immune persistence was not measured.

In this study, the trivalent formulation induced binding and neutralizing antibodies against all three vaccine components and protected mice against homologous challenge with each, despite the lower per-antigen dose. The trivalent vaccine combined the ancestral ERAGEM strain with the Delta and Omicron (BA.5) variants. After two doses, K18-hACE2 mice raised variant-specific binding and neutralizing antibodies against all three components and were protected against challenge with each vaccine-matched component: survival following lethal challenge with ERAGEM and Delta and, for BA.5, which was not lethal in unvaccinated controls, a reduction in viral burden. Infectious virus was undetectable in the lungs, and viral loads in the nasal turbinates were sharply reduced. Each antigen was present at just 1 µg per dose, against 1.5 µg in our bivalent vaccines [15] and 3–6 µg in the monovalent vaccine [12]. These are separate studies on one platform, not a single dose-titration, so we read the comparison cautiously; even so, protection and post-boost ancestral neutralizing titres were maintained at the lower per-antigen dose, an observation we treat as hypothesis-generating rather than as evidence of a dose-sparing effect.

We chose the three components to span the antigenic range of the pandemic rather than minor variations on a single antigen. ERAGEM anchors the formulation to the ancestral baseline; Delta adds a highly transmissible, virulent lineage that was uniformly lethal in our unvaccinated controls [2,19]; and BA.5 represents the immune-evasive Omicron sublineage that drove a major infection wave [3].

Antigenic competition, in which combining antigens weakens the response to one or more components, is a central concern in multivalent design. We did not test for antigenic competition directly, and in the absence of contemporaneous monovalent controls interference cannot be excluded; the preserved responses to all three components provide no indirect evidence of substantial interference under the conditions tested. Every component was boosted in both binding and neutralizing antibodies, all three challenge groups survived with infectious virus undetectable in the lungs, and the neutralizing response to BA.5 was in fact the strongest of the three (Table 1). Why the BA.5 component drew the strongest neutralizing response is not clear; it may reflect intrinsic differences in the immunogenicity of the vaccine antigens or assay-specific factors, and it warrants further study. Multivalent SARS-CoV-2 vaccines built on mRNA [6,20], spike or RBD subunit [7,21], virus-like particle [8], and viral-vectored [9,22] platforms have reported the same pattern: broader cross-reactivity without loss of component-specific immunity.

The protection observed at the reduced antigen dose may relate to several characteristics of the platform. A whole-virion antigen presents the entire structural-protein repertoire (spike, nucleocapsid, membrane, and envelope), not spike alone, widening the pool of available epitopes, some of them conserved across variants. The aluminium hydroxide adjuvant also favours durable antibody responses to particulate antigens. A formal dose-de-escalation study was not performed, so the dose-sparing interpretation should be regarded as exploratory. Even so, the pattern suggests that dividing a fixed antigen budget among more variants need not cost per-component protection, a useful trait for inactivated vaccines, where antigen yield constrains manufacturing.

These findings suggest a dose-sparing potential that warrants formal evaluation: covering additional variants may not require proportional increases in antigen content when whole-virion inactivated vaccines are used. Dedicated dose-comparison studies will be required to test it. If it holds, reducing the per-antigen dose to 1 µg could increase the number of doses obtainable from a fixed antigen yield, with potential manufacturing benefits.

We chose a whole-virion inactivated design over a nucleic-acid or subunit approach, though our group has worked on subunit antigens too, including a plant-produced receptor-binding-domain protein that gave strong neutralizing responses in mice [23]. The inactivated platform has practical strengths here. Its manufacturing is well established and already used at scale, and the product is stable under ordinary refrigeration, with no ultra-cold chain to maintain, an advantage where infrastructure is limited. Our antigens came from locally isolated strains made on the same platform as the licensed vaccine TURKOVAC, which has cleared clinical trials and authorization [12,13,14] and induces durable humoral and cellular immunity in people [24]. That gives us a validated production route and a realistic path to clinical testing of multivalent versions.

The closest prior study is that of Zhang and colleagues, whose inactivated trivalent vaccine (ancestral plus Delta and an early Omicron) broadened neutralizing antibodies in mice [17]. While that work primarily evaluated immunogenicity, the present study additionally assessed protection against homologous challenge and respiratory viral burden. It also used the later, more immune-evasive BA.5 sublineage and antigens from locally isolated strains, indicating that multivalent inactivated vaccines can be produced within a region. Trivalent inactivated coronavirus vaccines have likewise proved workable in veterinary use [16].

SARS-CoV-2 has kept evolving past BA.5, and current work targets XBB- and JN.1-lineage subvariants and broadly protective designs [25,26,27], including pan-sarbecovirus and pan-betacoronavirus strategies [10,11,28]. Our vaccine does not cover these newest subvariants and would need updating to match circulating strains. Beyond the specific antigen mix, it shows that a scalable inactivated platform can carry three antigens at a reduced per-antigen dose while protecting against each, and can be updated as dominant variants shift.

Because the trivalent formulation was not compared with contemporaneous monovalent or bivalent preparations, this study cannot directly establish a statistical advantage of the multivalent strategy over lower-valency counterparts. A complete experimental design should not be mistaken for demonstrated superiority. The comparison with our earlier monovalent (3–6 µg per antigen) and bivalent (1.5 µg per antigen) studies spans independent experiments performed at different times, and differences in virus stocks, antigen preparations, and assay runs cannot be excluded as contributors. In particular, the immunogenicity of the trivalent formulation was not compared head-to-head with that of the corresponding monovalent vaccines, so we cannot determine whether the response to any individual component differs from the response that component would elicit on its own. Such a comparison would require contemporaneous monovalent groups and remains the most direct way to test for antigenic interference; we regard it, together with the sequential-immunization experiment described below, as the priority for the follow-up study.

A further consideration specific to multivalent formulations containing historical strains is immune imprinting, or antigenic seniority, whereby prior exposure to an earlier antigen biases subsequent responses back toward it. This phenomenon has been described for SARS-CoV-2 and contributed to the decision to move from bivalent to monovalent mRNA boosters matched to circulating Omicron sublineages [4]. Our design cannot address this, and we note it as a limitation. The mice were antigenically naive, and testing imprinting directly would require a sequential-immunization design, in which animals are primed with one antigen and subsequently exposed to another; no such design was used here. We therefore cannot evaluate the contribution of imprinting to the responses we report, and we make no claim about how this formulation would behave in a population with pre-existing SARS-CoV-2 immunity. This is an important caveat to any translational reading of our results, and it is one reason we present the antigen combination used here as a proof of concept for the platform rather than as a formulation intended for current use.

Several mechanisms could in principle produce antigenic interference that our design would not detect. Co-adsorption of three whole virions onto the same aluminium hydroxide particles could mask epitopes; the components could compete for antigen presentation and for T-cell help; and immunodominance could drift toward the most immunogenic component. The disproportionately strong neutralizing response against BA.5 relative to the ancestral and Delta components is, in fact, compatible with such an immunodominance effect, although it may equally reflect intrinsic differences in the neutralization sensitivity of the challenge viruses or assay-specific factors. Indirect evidence of the kind presented here, namely that all three components elicited responses, cannot rule these mechanisms out; only contemporaneous monovalent controls can.

The formulation used a fixed dose of aluminium hydroxide (250 µg) for the combined antigen, prepared with the same standardized adsorption protocol used for our licensed inactivated vaccine, in which adsorption is a routinely controlled manufacturing parameter. We did not, however, measure the component-resolved adsorption efficiency of each of the three whole-virion antigens in this trivalent formulation, either individually or after mixing. If adsorption differs between components, the effective immunogenic dose of each may not correspond exactly to its nominal 1 µg, which bears on both the comparability of the three components and the interpretation of the dose-sparing observation. We therefore regard the dose-sparing finding as exploratory, and component-resolved adsorption and potency assays are a priority for the follow-up work.

Viral burden was assessed at a single time point, two days after challenge, the time point used in our previous challenge studies with this model, which allows comparison across them. It does, however, mean that our data describe viral burden at a single early time point rather than the kinetics of clearance, and we have avoided kinetic language accordingly. We regard survival and reduction in viral burden as complementary indicators of protection rather than a strict hierarchy. Survival following lethal challenge is the definitive outcome for the ERAGEM and Delta components, while the absence of detectable infectious virus in the lungs and the reduction in viral burden in the nasal turbinates are protective outcomes in their own right, although transmission itself was not assessed in this study. For the BA.5 component, where the challenge was not lethal, viral burden is the readout by which protection is assessed, and the protection observed should be understood as virological; body weight and temperature are reported as supporting clinical measures.

Multivalent strategies can be pursued on any platform, and the choice involves trade-offs. Nucleic-acid platforms are re-matched to a new strain most rapidly and tend to elicit Th1-biased responses, but require complex manufacturing and, for mRNA, stringent cold-chain conditions. Subunit and virus-like-particle approaches allow precise antigen design but depend heavily on adjuvant selection and present a narrower epitope repertoire. Aluminium-adjuvanted whole-virion inactivated vaccines, including the one used here, present the full structural-protein complement of the virion and are compatible with existing manufacturing lines and 2–8 °C storage, but are associated with Th2-skewed responses, are adjuvant-dependent, and have shown lower effectiveness than mRNA vaccines in several real-world settings. This does not mean that the inactivated platform is ineffective: inactivated whole-virion vaccines have nonetheless provided substantial real-world protection against severe disease: in a large population-based study conducted during an Omicron BA.2 wave, three doses of either BNT162b2 or CoronaVac conferred approximately 98% protection against severe outcomes [29]. We do not claim that the inactivated platform is immunologically superior; its advantage in the multivalent context is that an additional component can be added without altering the established production process.

Several specific obstacles would have to be resolved before a trivalent version of this platform could enter clinical development. First, the antigen ratio requires justification and optimisation: equal mass ratios were used here, but differing particle yields, adsorption behaviour, and intrinsic immunogenicity may argue for unequal ratios. Second, batch-to-batch consistency standards and release specifications must be established for a three-component drug substance, which requires component-resolved potency and identity assays rather than a single total-protein measurement. Third, adjuvant compatibility and the adsorption process must be validated for the mixed antigen. Fourth, the clinical endpoint requires careful selection: with field efficacy trials increasingly difficult to conduct, immunobridging against an approved comparator may be the more feasible path, yet the choice of comparator and of an accepted correlate of protection for multivalent inactivated vaccines remains unsettled.

This study has clear limits. Lung histopathology was not assessed, and we acknowledge this as a limitation. Histopathological evaluation would have provided information on residual inflammation and tissue injury that survival and viral-burden measurements cannot capture, and its absence limits what can be said about the quality of protection at the tissue level. The absence of detectable infectious virus in the lungs of vaccinated mice is consistent with limited pulmonary pathology, but does not establish it, since inflammatory changes can persist in the absence of recoverable infectious virus; histological confirmation is therefore necessary and is planned for future work. We did not measure cellular responses (for example, IFN-γ ELISPOT or CD4/CD8 intracellular cytokine staining) or mucosal immunity, so the immunogenicity assessment was restricted to serum binding and neutralizing antibodies. Because T-cell and mucosal responses were not measured, the protection observed cannot be attributed to humoral immunity alone; the antibody titres reported here accompanied protection but were not established as its mechanism, and the mechanistic interpretation of these findings is limited accordingly. These are planned follow-ups. The platform has engaged these arms in other studies, in that the monovalent vaccine gave moderate cellular responses preclinically [12], and TURKOVAC produced durable humoral and cellular immunity for up to eight months in vaccinees [24]. However, those findings cannot be extrapolated to the present trivalent formulation. Because protection was maintained despite a substantial reduction in the per-antigen dose, future work will examine how T-cell responses and immune memory contribute to this dose-sparing potential. Challenge was homologous to each component: the objective was not to assess heterologous breadth but to determine whether including three antigenically distinct whole-virion antigens at a reduced per-antigen dose compromised protection against any individual component. Heterologous challenge with non-included subvariants such as XBB or JN.1 is an obvious next step. Finally, this was a single trivalent group with few animals per arm in a mouse model, although the key outcomes (survival, lung clearance, and antibody boosting) were consistent across all animals. Clinical translation will need human studies, where factors such as age shape the response [30], but TURKOVAC’s clinical progression [13,14] offers a plausible route for multivalent versions on the same platform.

## 5. Conclusions

A whole-virion inactivated trivalent vaccine combining the ancestral (ERAGEM), Delta, and Omicron (BA.5) SARS-CoV-2 antigens elicited variant-specific humoral immunity and protected K18-hACE2 mice against challenge with each vaccine-matched component: against lethal challenge for ERAGEM and Delta, and against viral burden for Omicron BA.5, which did not cause mortality in unvaccinated controls. Infectious virus was undetectable in the lungs and viral burden in the nasal turbinates was reduced. These outcomes were obtained at a reduced per-antigen dose relative to our previously reported monovalent and bivalent formulations, which were independent studies rather than contemporaneous comparators; the observation therefore suggests a dose-sparing potential that warrants formal evaluation rather than establishing one. The study shows that a whole-virion inactivated platform can accommodate three antigenically distinct SARS-CoV-2 antigens while maintaining protection against each vaccine-matched component under the conditions tested. It does not establish an advantage over monovalent or bivalent formulations, which were not tested contemporaneously, and it does not address protection against variants absent from the formulation. The antigen combination used here is a proof of concept for the platform rather than a formulation proposed for current use.

## 6. Patents

Aykut Ozdarendeli, Shaikh Terkis Islam Pavel, Hazel Yetiskin, and Muhammet Ali Uygut are named inventors on patent applications covering inactivated SARS-CoV-2 vaccine development.

## Figures and Tables

**Table 1:** Variant-specific immune responses and protection elicited by the trivalent vaccine. For each component (also the homologous challenge variant), geometric mean titers (GMTs) of neutralizing (MN50) and binding (endpoint ELISA) antibodies are given after the first (prime) and the booster (boost) dose, with the corresponding fold-rise, alongside survival and pulmonary infectious-virus status after challenge. Challenge was lethal in unvaccinated controls for the ERAGEM and Delta components but not for Omicron BA.5, so survival in the BA.5 sub-group does not indicate protection against lethal disease. Each antigen was present at 1 µg per dose.

Component (Challenge Variant)	MN50 GMT (Prime → Boost)	Fold-Rise	ELISA GMT (Prime → Boost)	Fold-Rise	Survival	Lung Virus
ERAGEM	14.6 → 152.2	10.4×	3489.6 → 16,599.5	4.8×	100%	Not detected
Delta	12.3 → 128.0	10.4×	5868.8 → 27,916.9	4.8×	100%	Not detected
Omicron BA.5	64.0 → 609.8	9.5×	3805.4 → 16,566.9	4.4×	100%	Not detected

GMT, geometric mean titer; MN50, 50% microneutralization titer; ELISA, endpoint enzyme-linked immunosorbent assay titer. Prime, after the first immunization; boost, after the booster. Fold-rise is the boost-to-prime ratio. The fold-rise was closely matched across the three antigens (≈10× for neutralizing and ≈4.5× for binding antibodies), with broadly preserved component-specific responses across all three antigens; all prime-to-boost increases were statistically significant (Figure 4 and Figure 5). “Not detected” denotes no infectious virus recovered from lung homogenates by the focus-forming assay two days post-challenge.

## Data Availability

The raw data supporting the conclusions of this article will be made available by the corresponding author on request.
